# Prediction of the risk of developing end-stage renal diseases in newly diagnosed type 2 diabetes mellitus using artificial intelligence algorithms

**DOI:** 10.1186/s13040-023-00324-2

**Published:** 2023-03-10

**Authors:** Shuo-Ming Ou, Ming-Tsun Tsai, Kuo-Hua Lee, Wei-Cheng Tseng, Chih-Yu Yang, Tz-Heng Chen, Pin-Jie Bin, Tzeng-Ji Chen, Yao-Ping Lin, Wayne Huey-Herng Sheu, Yuan-Chia Chu, Der-Cherng Tarng

**Affiliations:** 1 Division of Nephrology, Department of Medicine, Taipei Veterans General Hospital, 201, Section 2, Shih-Pai Road, Taipei, 11217 Taiwan; 2 School of Medicine, College of Medicine, National Yang Ming Chiao Tung University, Taipei, Taiwan; 3 Institute of Clinical Medicine, National Yang Ming Chiao Tung University, Taipei, Taiwan; 4 Graduate Institute of Medicine, College of Medicine, Kaohsiung Medical University, Kaohsiung, Taiwan; 5 Department of Family Medicine, Taipei Veterans General Hospital, Taipei, Taiwan; 6 Department of Family Medicine, Taipei Veterans General Hospital, Hsinchu Branch, Hsinchu, Taiwan; 7 Institute of Hospital and Health Care Administration, National Yang Ming Chiao Tung University, Taipei, Taiwan; 8 Division of Endocrinology and Metabolism, Department of Internal Medicine, Taipei Veterans General Hospital, Taipei, Taiwan; 9 Institute of Molecular and Genetic Medicine, National Health Research Institute, Miaoli, Taiwan; 10 Information Management Office, Taipei Veterans General Hospital, 201, Section 2, Shih-Pai Road, Taipei, 11217 Taiwan; 11 Big Data Center, Taipei Veterans General Hospital, Taipei, Taiwan; 12 Department of Information Management, National Taipei University of Nursing and Health Sciences, Taipei, Taiwan; 13 Department and Institute of Physiology, National Yang Ming Chiao Tung University, Taipei, Taiwan

**Keywords:** Artificial intelligence, Diabetes mellitus, End-stage renal disease, Machine learning

## Abstract

**Objectives:**

Type 2 diabetes mellitus (T2DM) imposes a great burden on healthcare systems, and these patients experience higher long-term risks for developing end-stage renal disease (ESRD). Managing diabetic nephropathy becomes more challenging when kidney function starts declining. Therefore, developing predictive models for the risk of developing ESRD in newly diagnosed T2DM patients may be helpful in clinical settings.

**Methods:**

We established machine learning models constructed from a subset of clinical features collected from 53,477 newly diagnosed T2DM patients from January 2008 to December 2018 and then selected the best model. The cohort was divided, with 70% and 30% of patients randomly assigned to the training and testing sets, respectively.

**Results:**

The discriminative ability of our machine learning models, including logistic regression, extra tree classifier, random forest, gradient boosting decision tree (GBDT), extreme gradient boosting (XGBoost), and light gradient boosting machine were evaluated across the cohort. XGBoost yielded the highest area under the receiver operating characteristic curve (AUC) of 0.953, followed by extra tree and GBDT, with AUC values of 0.952 and 0.938 on the testing dataset. The SHapley Additive explanation summary plot in the XGBoost model illustrated that the top five important features included baseline serum creatinine, mean serum creatine within 1 year before the diagnosis of T2DM, high-sensitivity C-reactive protein, spot urine protein-to-creatinine ratio and female gender.

**Conclusions:**

Because our machine learning prediction models were based on routinely collected clinical features, they can be used as risk assessment tools for developing ESRD. By identifying high-risk patients, intervention strategies may be provided at an early stage.

**Supplementary Information:**

The online version contains supplementary material available at 10.1186/s13040-023-00324-2.

## Introduction

Type 2 diabetes mellitus (T2DM) is a major challenge to public health worldwide, and the assessment and management of this chronic disease impose a heavy economic burden [1, 2]. T2DM is associated with many complications and problematic symptoms, including micro- and macrovascular complications [3, 4]. Among these complications, diabetic kidney disease (DKD) is a leading cause of chronic kidney disease (CKD) and is associated with a future risk of progression to end-stage renal disease (ESRD) [5, 6]. However, a diagnosis of DKD is often delayed, particularly in the early stages of the disease, because most patients remain asymptomatic with respect to kidney dysfunction [7]. Therefore, identifying DKD patients with a rapid decline in the estimated glomerular filtration rate (eGFR) might be helpful for allowing early nephroprotective treatment to be administered to delay or prevent the progression of kidney failure.

Previous large-scale population-based cohort studies have identified multiple factors potentially contributing to rapid eGFR decline, such as hypertension [8, 9], proteinuria [10], demographic factors, and underlying comorbidities [7]. A meta-analysis of demographic and clinical laboratory data from twenty cohorts representing 41,271 T2DM patients was conducted to develop a categorization point system for DKD prediction [11]. The prediction model achieved an average area under the receiver operating characteristic curve (AUC) of 0.765. Because electronic health record usage provides hundreds of clinical features and a large volume of data, prediction models using a categorization point system may be insufficient to effectively make use of unaligned and correlated data structures. Recently, artificial intelligence (AI) has changed modern procedures, and the progress of machine learning with big data analysis has improved the capacity of predictive model development [12].

In a cohort study consisting of diabetic patients, an AI model using logistic regression was developed to predict the progression of DKD according to 3073 features [13], and it achieved an AUC of 0.743 and an average accuracy of 71%. However, only logistic regression was applied in this study, and the predictive ability of other machine learning models with respect to renal function progression in diabetic patients remains unknown. In addition, in the abovementioned study, the AI model predicted DKD progression for 6 months after the enrollment period; therefore, its predictive ability for a longer follow-up period is unknown.

In our study, we used a large-scale newly diagnosed DM cohort to perform machine learning models by using clinical features, including demographic characteristics, comorbidities, laboratory data and concomitant medications from outpatient department and emergency room visits as well as hospital admissions, to predict the risks of developing ESRD with a long follow-up period. We also used SHapley Additive exPlanation (SHAP) values to evaluate the accurate attribution values for each important feature within machine learning models.

## Methods

### Data sources and study population

During the period of January 2008 to December 2018, we constructed a T2DM 10-year retrospective longitudinal cohort based on the information of patients with newly diagnosed T2DM from the Big Data Center, which includes the detailed patient demographic, underlying comorbidities, medication prescriptions, and laboratory data from all inpatient, outpatient and emergency services [14]. Patients without at least two eGFR values were excluded from our analyses. In addition, we excluded T2DM patients who had undergone renal replacement therapy, such as hemodialysis, peritoneal dialysis, and kidney transplant, before the enrollment points. This study was approved by the Institutional Review Board (Taipei Veterans General Hospitals, Approval no. 2022–03-006 AC), and the need for informed consent was waived because the data were deidentified.

### Feature selection

We extracted 78 features used for machine learning, including demographic characteristics, underlying comorbidities, laboratory data and concomitant drugs. The demographic characteristics included age, gender, smoking and alcohol consumption. Underlying comorbidities included histories of hypertension, transient ischemic attack, ischemic stroke, hemorrhagic stroke, myocardial infarction, coronary artery disease, congestive heart failure, chronic liver disease, cirrhosis, peptic ulcer disease, autoimmune disease, chronic obstructive pulmonary disease, asthma, peripheral arterial occlusive disease, cancer, gout, atrial fibrillation, valvular heart disease and diabetic retinopathy. The laboratory data included baseline serum creatinine, mean serum creatinine assessed within 1 year before the diagnosis of T2DM, cholesterol, triglycerides, low-density lipoprotein cholesterol, high-density lipoprotein cholesterol, uric acid, calcium, phosphate, white blood cells, hemoglobin, albumin, alanine aminotransferase, aspartate aminotransferase, total bilirubin, direct bilirubin, alkaline phosphatase, gamma-glutamyl transferase, glycated hemoglobin, glucose, the international normalized ratio, activated partial thromboplastin time, high-sensitivity C-reactive protein, iron, thyroid-stimulating hormone, free thyroxine, and spot urine protein-to-creatinine ratio (UPCR). Concomitant medications included renin-angiotensin-aldosterone system (RAAS) inhibitors, alpha blockers, beta blockers, calcium channel blockers, warfarins, direct oral anticoagulants, aspirins, clopidogrels, nitrates, statins, diuretics, spironolactones, metformins, sulfonylureas, meglitinides, sodium–glucose cotransporter 2 inhibitors, glucagon-like peptide-1 receptor agonists, dipeptidyl peptidase-4 inhibitors, thiazolidinediones, alpha-glucosidase inhibitors, insulins, nonsteroidal anti-inflammatory drugs, cyclooxygenase-2 inhibitors, proton pump inhibitors, steroids, allopurinols, febuxostats and benzbromarones.

### Class definition

In our study, the class was annotated as 1 if there was ESRD occurrence during the follow-up periods (defined as eGFR < 15 ml/min/1.73 m^2^ or the receipt of maintenance dialysis or kidney transplant), and the class was annotated as 0 if there was no ESRD occurrence. We calculated eGFR using the Chronic Kidney Disease Epidemiology Collaboration (CKD-EPI) equations [15].

### Data cleaning and machine learning model development

Categorical variables are presented as numbers (proportions) and continuous parametric variables are shown as the median (interquartile ranges [IQRs]). To impute the missing values of the clinical features, the K-nearest neighbor (KNN) algorithm was used before the machine learning methods [16, 17]. For model development, the study cohort was randomly divided to create a 70%:30% training set to test set ratio. Because the number of ESRD cases was much smaller than the number of non-ESRD cases, we performed the synthetic minority over-sampling technique (SMOTE)-Tomek algorithms to balance the number of samples taken for imbalanced data [18, 19]. Six machine learning models, including logistic regression, extra trees [20], random forest [21], gradient boosting decision tree (GBDT) [22], extreme gradient boosting models (XGBoost) [23], and light gradient boosting machine (LGBM) [24], are performed. We used forward-feature selection for the reduction in dimensions, which selects the most useful subset of features from all available features [25, 26]. Five-fold cross-validation is performed on the training set to estimate the performance and validate the stability of the applied machine learning models [27, 28].

### Hyperparameter optimization

A grid search in combination with the five-fold cross-validation was conducted to optimize the hyperparameters of logistic regression, extra trees, random forest, GBDT, XGBoost, and LGBM to achieve the best F1 score [29–31]. The details of hyperparameter optimization for each ensemble model are listed in Table 1. Grid searches determine the best hyperparameter value based on a set of given values.

**Table 1:** Hyperparameters of machine learning models

Model	Hyperparameters	Range	Optimal values
**Logistic regression**	penalty	[l1, l2]	l2
	Cs	[0.001, 0.1, 1, 100, 1000]	1
**Extra trees**	min_samples_leaf	[5, 8, 10]	5
	criterion	[gini, entropy, log_loss]	entropy
	max_features	[sqrt, log2, none]	sqrt
**Random forest**	max_depth	[3, 5, 10]	10
	min_samples_split	[2, 5, 10]	5
**GBDT**	learning_rate	[0.01, 0.1, 0.2]	0.2
	max_depth	[3, 5, 8]	8
	n_estimators	[10, 20]	20
**XGBoost**	gamma	[0.5, 1, 5]	0.5
	colsample_bytree	[0.6, 0.8, 1.0]	1.0
	max_depth	[3, 4, 5]	5
**LGBM**	n_estimators	[8, 16, 24]	24
	num_leaves	[6, 12, 16]	16
	max_bin	[255, 510]	510

*Abbreviation*s: *GBDT* gradient boosting decision tree, *XGBoost* extreme gradient boosting, *LGBM* light gradient boosting machine

### Model evaluation

The discriminative abilities of the different machine learning models were compared based on their AUCs. In addition, the F1 score, accuracy, precision, recall, average precision and log loss values of each model by using testing dataset were also presented. SHapley Additive exPlanations (SHAP) was used to evaluate the risk of developing ESRD in T2DM and to provide explanations for the attribution values of clinical features in a unified framework to interpret model predictions.

### Software and package applicating for modeling

We used Python (Python Software Foundation version 3.7.6, available at http://www.python.org) and open-source Scikit-learn library for the establishment of machine learning models and SAS version 9.4 (SAS Institute, Cary, NC) for statistical analysis [32]. We used Python and Scikit-learn library packages, including sklearn.impute.KNNImputer for missing value imputation, sklearn.model_selection.train_test_split for randomly dividing data into train and test sets, sklearn.model_selection.GridSearchCV for hyperparameter optimization, sklearn.linear_model.LogisticRegression for development of the logistic regression model, sklearn.ensemble.ExtraTreesClassifier for development of the extra tree model, sklearn.ensemble.RandomForestClassifier for development of the random forest model, sklearn.ensemble.GradientBoostingClassifier for development of the GBDT model, XGBoost Python package for development of the XGBoost model, lightgbm.LGBMClassifier Python package for development of the LGBM model, and sklearn.model_selection.StratifiedKFold for cross-validation. A *P* value of 0.05 was considered statistically significant.

## Results

### Characteristics and distribution of patients

A total of 105,234 T2DM patients aged > 20 years old were identified during the 10-year study period, of whom 34,059 had no eGFR measurements, 16,351 did not have at least two eGFR values, and 1347 patients receiving renal replacement therapy were excluded, which resulted in a final cohort of 53,477 T2DM patients. The detailed patient demographic data are provided in Table 2. The median patient age was 67.05 years (IQR 57.37 to 77.74 years), and 41.4% of the patients were female. In addition, 58.2% of patients had hypertension, 19.8% had coronary artery disease, and 23.4% had cancer. Regarding renal function, T2DM patients had baseline serum creatinine levels of 0.94 mg/dL (IQR 0.75 to 1.27 mg/dL), mean serum creatinine of 0.95 mg/dL (IQR 0.76 to 1.26 mg/dL) within 1 year before the diagnosis of T2DM. The dataset was randomly divided into a training set (70%) and a testing set (30%). Of all the T2DM patients, 4769 (8.9%) patients developed ESRD. A total of 3334 (8.9%) patients developed ESRD on the training set, and 1435 (8.9%) patients developed ESRD on the testing set.

**Table 2:** Demographics and clinical features between T2DM patients

	Full cohort	Training set	Testing set
	(***n*** = 53,477)	(***n*** = 37,433)	(***n*** = 16,044)
**Demographic data**			
**Age, years**	67.05 [57.37, 77.74]	67.09 [57.46, 77.78]	66.97 [57.20, 77.66]
**Female gender,*n*(%)**	22,162 (41.4)	15,508 (41.4)	6654 (41.5)
**Smoking,*n*(%)**	12,424 (23.2)	8690 (23.2)	3734 (23.3)
**Alcohol consumption,*n*(%)**	9117 (17.0)	6438 (17.2)	2679 (16.7)
**Underlying comorbidities**			
**Hypertension,*n(*%)**	31,142 (58.2)	21,816 (58.3)	9326 (58.1)
**Transient ischemic attack,*n*(%)**	541 (1.0)	371 (1.0)	170 (1.1)
**Ischemic stroke,*n*(%)**	3359 (6.3)	2339 (6.2)	1020 (6.4)
**Hemorrhagic stroke,*n*(%)**	758 (1.4)	533 (1.4)	225 (1.4)
**Myocardial infarction,*n*(%)**	1806 (3.4)	1251 (3.3)	555 (3.5)
**Coronary artery disease,*n*(%)**	10,585 (19.8)	7449 (19.9)	3136 (19.5)
**CHF,*n*(%)**	3032 (5.7)	2139 (5.7)	893 (5.6)
**Chronic liver disease,*n*(%)**	4377 (8.2)	3085 (8.2)	1292 (8.1)
**Cirrhosis,*n*(%)**	1193 (2.2)	859 (2.3)	334 (2.1)
**Peptic ulcer disease,*n*(%)**	4613 (8.6)	3238 (8.7)	1375 (8.6)
**Autoimmune disease,*n*(%)**	508 (0.9)	360 (1.0)	148 (0.9)
**COPD,*n*(%)**	2881 (5.4)	2026 (5.4)	855 (5.3)
**Asthma,*n*(%)**	1132 (2.1)	784 (2.1)	348 (2.2)
**PAOD,*n*(%)**	99 (0.2)	74 (0.2)	25 (0.2)
**Cancer,*n*(%)**	12,513 (23.4)	8788 (23.5)	3725 (23.2)
**Gout,*n*(%)**	2445 (4.6)	1751 (4.7)	694 (4.3)
**Atrial fibrillation,*n*(%)**	1589 (3.0)	1133 (3.0)	456 (2.8)
**Valvular heart disease,*n*(%)**	1311 (2.5)	917 (2.4)	394 (2.5)
**Diabetic retinopathy,*n*(%)**	2744 (5.1)	1911 (5.1)	833 (5.2)
**Laboratory data at the diagnosis of T2DM**			
**Creatinine, mg/dL**			
**Baseline serum creatinine, mg/dL**	0.94 [0.75, 1.27]	0.94 [0.76, 1.27]	0.94 [0.75, 1.27]
**Mean serum creatinine, mg/dL**^a^	0.95 [0.76, 1.26]	0.95 [0.76, 1.26]	0.94 [0.76, 1.26]
**Cholesterol, mg/dL**	175.00 [155.00, 195.20]	175.00 [155.00, 195.00]	175.00 [155.95, 196.00]
**Triglyceride, mg/dL**	129.40 [94.00, 177.00]	130.00 [94.60, 177.00]	129.00 [94.00, 176.00]
**LDL-C, mg/dL**	104.00 [89.20, 120.00]	104.00 [89.00, 120.00]	104.40 [89.80, 120.40]
**HDL-C, mg/dL**	44.60 [39.00, 50.60]	44.60 [39.00, 50.60]	44.80 [39.00, 50.80]
**Uric acid, mg/dL**	5.98 [5.00, 7.00]	5.98 [5.00, 7.00]	5.96 [5.00, 7.00]
**Calcium, mg/dL**	9.18 [8.92, 9.40]	9.18 [8.92, 9.40]	9.16 [8.92, 9.40]
**Phosphate, mg/dL**	3.30 [3.04, 3.54]	3.30 [3.04, 3.54]	3.30 [3.04, 3.56]
**White blood cells, /mm**^**3**^	7300 [6100, 8860]	7300 [6100, 8860]	7300 [6100, 8820]
**Hemoglobin, g/dL**	12.60 [11.40, 13.70]	12.60 [11.40, 13.70]	12.60 [11.40, 13.70]
**Albumin, g/dL**	3.90 [3.58, 4.16]	3.90 [3.58, 4.16]	3.90 [3.56, 4.16]
**Alanine transaminase, U/L**	23.00 [17.00, 35.00]	23.00 [17.00, 35.00]	23.00 [17.00, 35.00]
**Aspartate transaminase, U/L**	23.20 [19.00, 31.00]	23.20 [19.00, 31.00]	23.20 [18.80, 31.00]
**Total bilirubin, mg/dL**	0.69 [0.51, 1.00]	0.69 [0.51, 1.00]	0.69 [0.51, 0.99]
**Direct bilirubin, mg/dL**	0.26 [0.17, 0.38]	0.26 [0.17, 0.38]	0.26 [0.17, 0.38]
**Alkaline phosphatase, U/L**	84.00 [71.20, 104.20]	84.00 [71.20, 104.00]	84.00 [71.00, 104.20]
**Gamma-glutamyl transferase, U/L**	43.00 [28.80, 73.60]	43.00 [28.80, 73.80]	43.40 [28.80, 73.40]
**HbA1c, %**	7.70 [6.90, 9.42]	7.70 [6.90, 9.43]	7.70 [6.88, 9.40]
**Glucose, mg/dL**	142.00 [117.00, 186.00]	142.00 [117.00, 186.00]	142.00 [117.00, 186.00]
**INR**	1.02 [0.99, 1.10]	1.02 [0.99, 1.10]	1.02 [0.99, 1.10]
**aPTT, seconds**	57.56 [48.96, 85.10]	57.56 [48.96, 85.10]	57.37 [48.96, 85.10]
**Hs-CRP, mg/dL**	1.08 [0.32, 1.52]	1.08 [0.32, 1.53]	1.08 [0.32, 1.43]
**Iron, μg/dL**	64.60 [54.00, 77.20]	64.60 [54.00, 77.00]	64.40 [53.80, 77.20]
**TSH, uIU/mL**	1.77 [1.22, 2.31]	1.76 [1.22, 2.31]	1.78 [1.22, 2.31]
**Free T4, ng/dL**	1.08 [1.01, 1.17]	1.08 [1.01, 1.17]	1.08 [1.01, 1.17]
**Spot urine protein-creatinine ratio, g/g**	2.28 [0.50, 3.43]	2.32 [0.51, 3.43]	2.19 [0.49, 3.41]
**Concomitant medications**			
**RAAS inhibitors,*n*(%)**	30,111 (56.3)	21,093 (56.3)	9018 (56.2)
**Alpha, blocker,*n*(%)**	14,189 (26.5)	9927 (26.5)	4262 (26.6)
**Beta blocker,*n*(%)**	21,922 (41.0)	15,333 (41.0)	6589 (41.1)
**CCB,*n*(%)**	27,749 (51.9)	19,465 (52.0)	8284 (51.6)
**Warfarin,*n*(%)**	1761 (3.3)	1234 (3.3)	527 (3.3)
**DOAC,*n*(%)**	138 (0.3)	94 (0.3)	44 (0.3)
**Aspirin,*n*(%)**	16,713 (31.3)	11,766 (31.4)	4947 (30.8)
**Clopidogrel, n(%)**	6709 (12.5)	4770 (12.7)	1939 (12.1)
**Nitrate,*n*(%)**	12,810 (24.0)	8960 (23.9)	3850 (24.0)
**Statin,*n*(%)**	22,656 (42.4)	15,843 (42.3)	6813 (42.5)
**Diuretic,*n*(%)**	18,660 (34.9)	13,073 (34.9)	5587 (34.8)
**Spironolactone,*n*(%)**	5066 (9.5)	3568 (9.5)	1498 (9.3)
**Metformin,*n*(%)**	37,396 (69.9)	26,158 (69.9)	11,238 (70.0)
**Sulfonylurea,*n*(%)**	25,202 (47.1)	17,631 (47.1)	7571 (47.2)
**Meglitinide,*n*(%)**	8625 (16.1)	6024 (16.1)	2601 (16.2)
**SGLT2 inhibitor,*n*(%)**	505 (0.9)	358 (1.0)	147 (0.9)
**GLP1 receptor agonist,*n*(%)**	78 (0.1)	60 (0.2)	18 (0.1)
**Dipeptidyl peptidase-4 inhibitor,*n*(%)**	16,164 (30.2)	11,299 (30.2)	4865 (30.3)
**Thiazolidinedione,*n*(%)**	4847 (9.1)	3408 (9.1)	1439 (9.0)
**Alpha-glucosidase inhibitor,*n*(%)**	8530 (16.0)	5956 (15.9)	2574 (16.0)
**Insulin,*n*(%)**	26,752 (50.0)	18,790 (50.2)	7962 (49.6)
**NSAID,*n*(%)**	26,349 (49.3)	18,450 (49.3)	7899 (49.2)
**COX-2 inhibitor,*n*(%)**	7230 (13.5)	5037 (13.5)	2193 (13.7)
**Proton pump inhibitor,*n*(%)**	14,700 (27.5)	10,267 (27.4)	4433 (27.6)
**Steroid,*n*(%)**	8747 (16.4)	6100 (16.3)	2647 (16.5)
**Allopurinol,*n*(%)**	2466 (4.6)	1711 (4.6)	755 (4.7)
**Febuxostat,*n*(%)**	1065 (2.0)	734 (2.0)	331 (2.1)
**Benzbromarone,*n*(%)**	4388 (8.2)	3078 (8.2)	1310 (8.2)

*Abbreviation*s: *T2DM* type 2 diabetes mellitus, *CHF* congestive heart failure, *COPD* chronic obstructive pulmonary disease, *PAOD* peripheral arterial occlusive disease, *LDL-C* low-density lipoprotein cholesterol, *HDL-C* high density lipoprotein-cholesterol, *HbA1c* glycated hemoglobin, *INR* international normalized ratio, *aPTT* activated partial thromboplastin time, *Hs-CRP* high-sensitivity C-reactive protein, *TSH* thyroid stimulating hormone, *T4* thyroxine, *RAAS* renin-angiotensin system, *CCB* calcium channel blocker, *DOAC* direct oral anticoagulant, *SGLT2* sodium-glucose cotransporter 2, *GLP1* glucagon-like peptide-1, *NSAID* nonsteroidal anti-inflammatory drug, *COX-2* cyclooxygenase-2

^a^ The mean serum creatinine value assessed within 1 year before the diagnosis of T2DM

### Model prediction ability

**Fig. 1:**
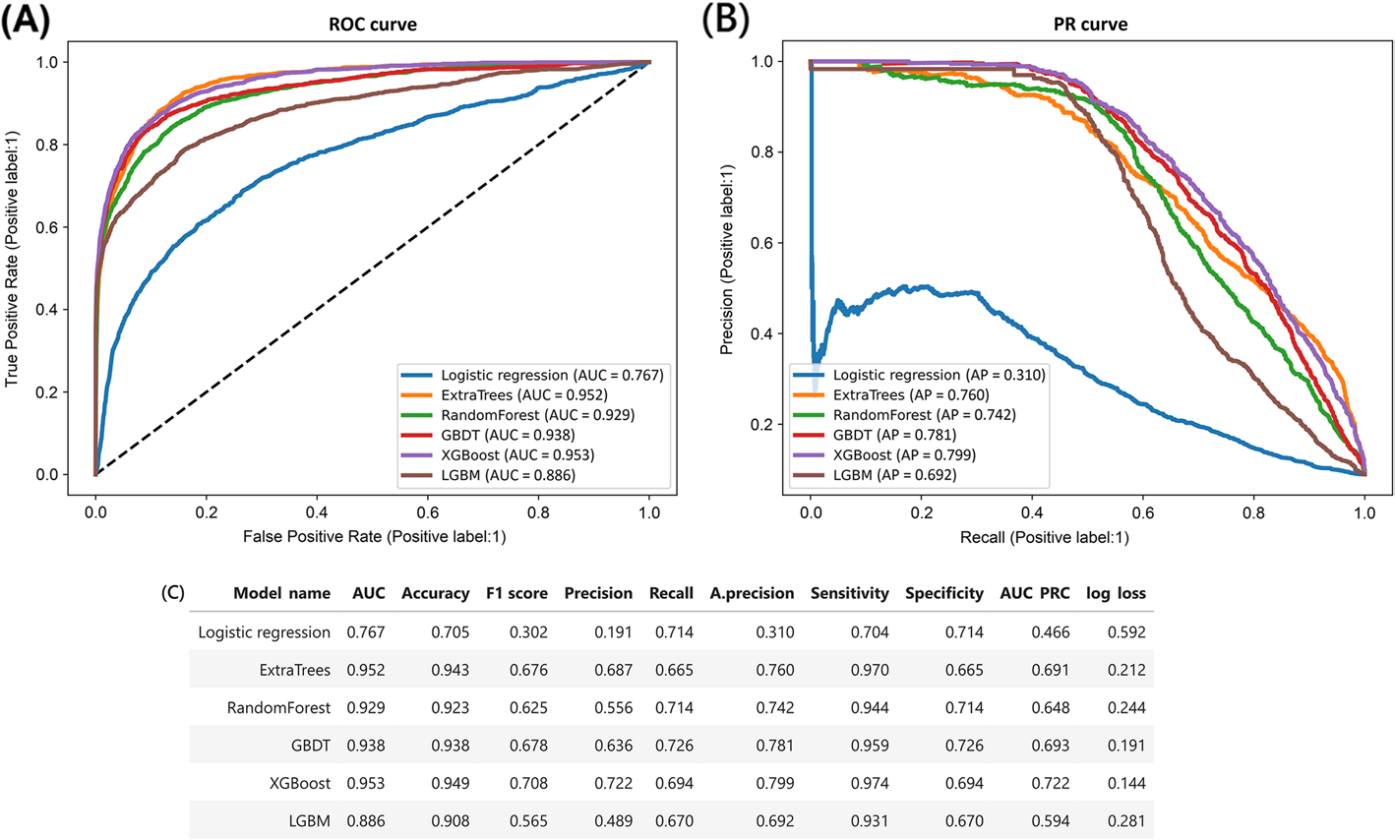
**A** Receiver operating characteristic curves and **B** precision–recall curves of machine learning models on the testing dataset. **C** XGBoost yielded the highest area under the ROC curve for prediction of end-stage renal disease followed by extra trees classifier and GBDT on the testing dataset. *Abbreviations*: ROC, receiver operating characteristic; PR, precision–recall; AUC, area under curve of receiver operating characteristic curve; A.precision, average precision; AUC PRC, area under curve of precision-recall curve; GBDT, gradient boosting decision tree; XGBoost, extreme gradient boosting; LGBM, light gradient boosting machine

Six machine learning models, i.e., logistic regression, extra tree classifier, random forest, GBDT, XGBoost, and LGBM, were performed, and the AUCs and other performance indices, such as accuracy, F1 score, precision, recall and average precision achieved by the machine learning models after data augmentation are presented in Supplementary Table 1. The AUCs resulting from 5-fold cross-validation of XGBoost models with a mean of 0.984 (Supplementary Fig. 1). On the testing dataset, AUCs showed that the XGBoost model had the highest predictive ability, with an AUC of 0.953, followed by the extra tree model with an AUC of 0.952 (Fig. 1).

### Ranks of feature importance and SHAP values in the XGBoost model

**Fig. 2:**
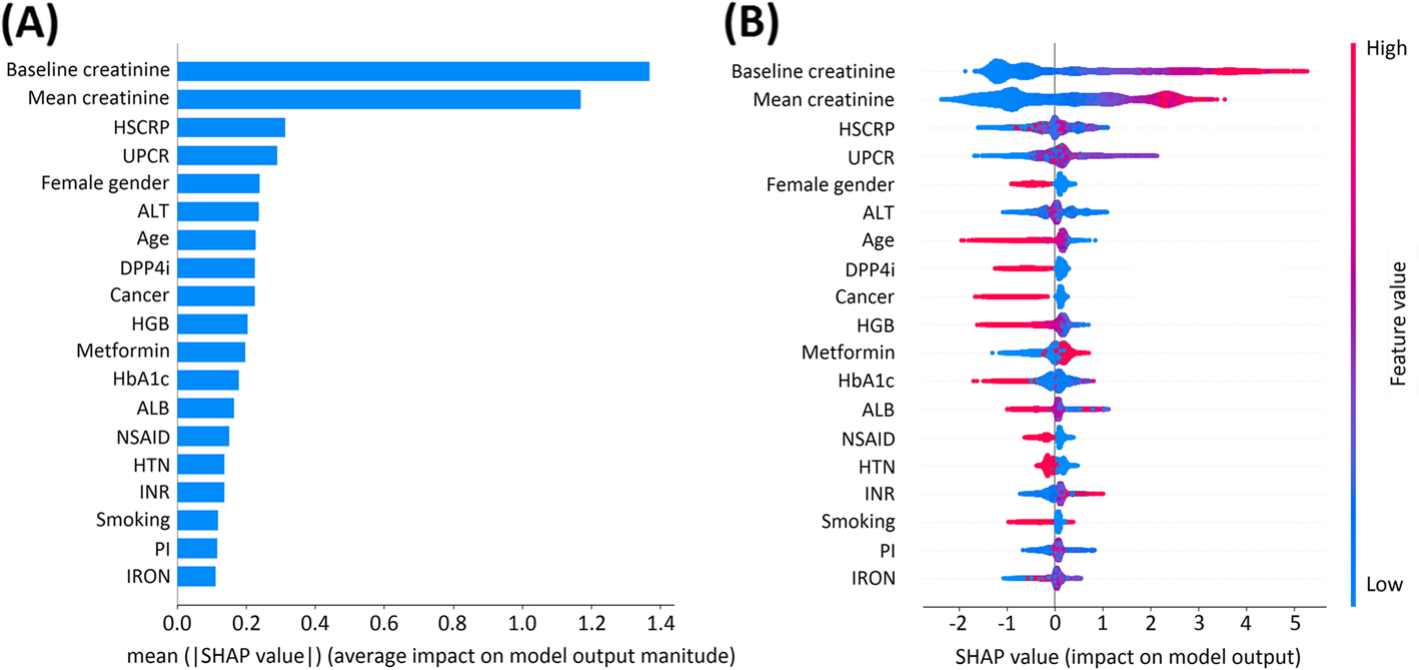
**A** The feature importance plot and **B** SHAP summary plot showed the top clinical important features for predicting risks of developing end-stage renal disease in the XGBoost model. *Abbreviations*: XGBoost, extreme gradient boosting; HSCRP, high-sensitivity C-reactive protein; UPCR, spot urine protein-to-creatinine ratio; ALT, alanine transaminase; DPP4i, dipeptidyl peptidase 4 inhibitors; HGB, hemoglobin; HbA1c, glycated hemoglobin; ALB, albumin; NSAID, nonsteroidal anti-inflammatory drug; HTN, hypertension; INR, international normalized ratio; PI, phosphate

We performed feature importance plots of the XGBoost model based on the SHAP values and listed the top important features sorted by the impacts in descending order (Fig. 2A). The top five important features were baseline serum creatinine, mean serum creatinine within 1 year before the diagnosis of T2DM, high-sensitivity C-reactive protein, UPCR and female gender. The impacts of feature importance on model output were also illustrated in the SHAP summary plot (Fig. 2B). Higher SHAP values of important features indicate a higher probability of impacts of the prediction in the XGBoost model. SHAP values in red dots indicate an increase in prediction, while those in blue dots indicate a decrease in prediction. Baseline serum creatinine, mean serum creatinine within 1 year before the diagnosis of T2DM, high-sensitivity C-reactive protein, and UPCR showed positive impacts on the prediction of developing ESRD risk, while the female gender showed a negative impact.

### The dependent plots of interactions between serum creatinine, high-sensitivity C-reactive protein, UPCR and female gender

**Fig. 3:**
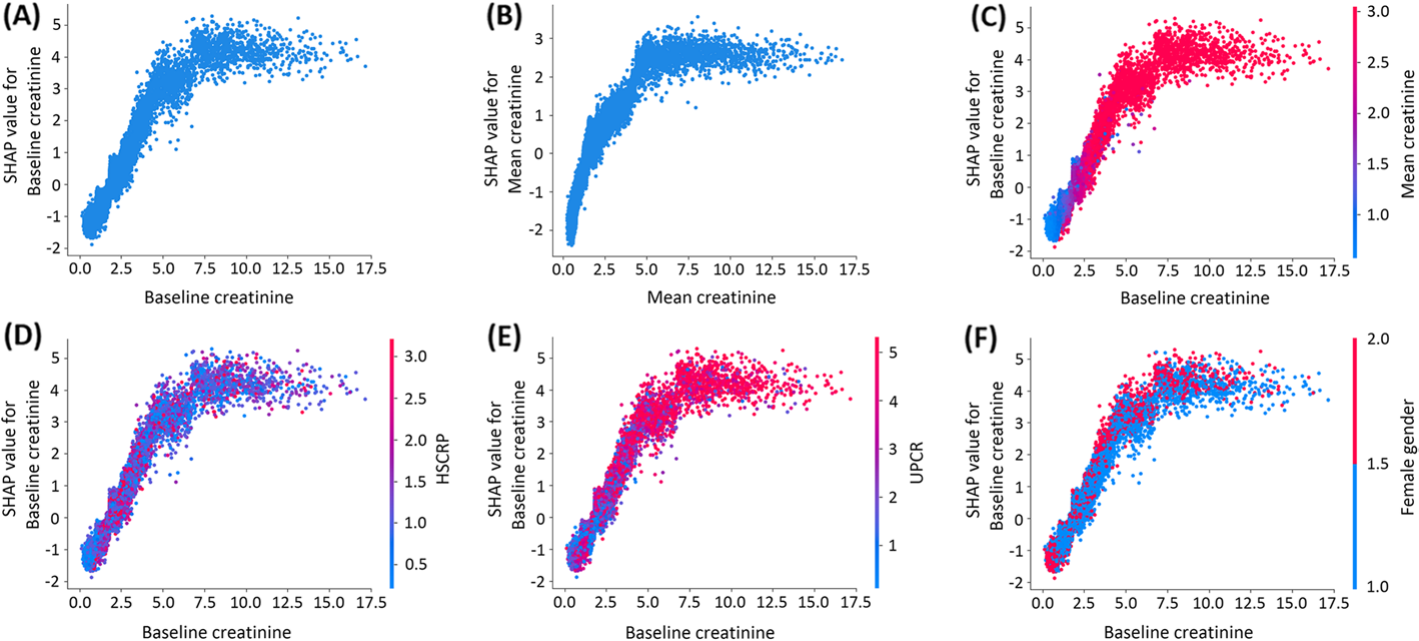
The plots of SHAP value of (**A**) baseline serum creatinine and (**B**) mean serum creatinine within 1 year before diagnosis showed increased creatinine levels were associated with increased SHAP values. SHAP interaction plots showed the interaction impacts between baseline serum creatinine and (**C**) mean serum creatinine (**D**) HSCRP, (**E**) UPCR, and (**F**) female gender on the prediction model’s output. *Abbreviations*: SHAP, SHapley Additive exPlanations; HSCRP, high-sensitivity C-reactive protein; UPCR, spot urine protein-creatinine ratio

As shown in Fig. 3, the dependent plots illustrated the SHAP values and the interactions between serum creatinine, high-sensitivity C-reactive protein, UPCR and female gender in the XGBoost model. The risks of developing ESRD increased as baseline or mean serum creatinine increased and then reached a plateau when creatinine > 5 mg/dL (Fig. 3A–B). Figure 3C–F illustrates the interaction between the SHAP values of baseline serum creatinine, mean serum creatinine, high-sensitivity C-reactive protein, UPCR and female gender. The values on the y-axis indicate the interaction SHAP values between baseline serum creatinine and other important features, and values on the x-axis are the levels of baseline serum creatinine. Mean serum creatinine, high-sensitivity C-reactive protein, UPCR and female gender were positively correlated with the predictive value of baseline serum creatinine.

## Discussion

In the current study, we developed machine learning models to predict the development of ESRD among T2DM patients based on electronic medical records. We used the machine learning system to conduct feature selection and compare the AUCs among the different machine learning models. We found that the XGBoost model had the highest predictive performance with the highest AUC of 0.953 on the testing dataset compared to other machine learning algorithms. The top five important features were baseline serum creatinine, mean serum creatinine within 1 year before the diagnosis of T2DM, high-sensitivity C-reactive protein, UPCR and female gender.

Previous studies in nondiabetic populations have attempted to find useful markers to predict ESRD. A Norwegian large-scale general health study including 65,589 adults aged > 20 years from 1995 through 1997 established a clinical predictive model (incorporating age, gender, physical activity, diabetes, systolic blood pressure, antihypertensive medication, and high-density lipoprotein) for the future risk of ESRD, and the AUC reached 0.864 [33]. After adding albuminuria and eGFR, the AUC of the model was increased to 0.936. Ishani et al [34]. studied 12,866 men who were at high risk for heart disease and found that dipstick proteinuria, eGFR < 60 ml/min/1.73 m^2^, and hematocrit were related to the development of ESRD. Because the study populations were limited to nondiabetic populations, the findings of these studies may not be generalizable to T2DM groups. For diabetic patients, proteinuria [35, 36], diabetic retinopathy [37, 38], increased glycated hemoglobin levels [39], hypertension [40], and cardiovascular diseases [41, 42] may precede kidney function decline and have been demonstrated to be associated with renal function progression.

A customized software program for CKD risk identification in Australia (the Electronic Diagnosis and Management Assistance to Primary Care in Chronic Kidney Disease (eMAP:CKD) program) was developed to integrate primary care electronic health records from more than 150,000 patients [43]. After the initiation of the program, there was a significant improvement in CKD documentation from 0.48 to 1.55%. In addition, the proportions of at-risk patients diagnosed with CKD at 15 months were found to be significantly increased from 7.8 to 24.40%. Furthermore, recent studies have applied AI to predict the risks of CKD. Kanda et al. [44] conducted a study including 7465 subjects and found that AI models with support vector machine (SVM) models can help predict CKD progression in both high-risk and low-risk subjects. After the 3-year follow-up, the accuracy of the SVM models was increased. Chen et al. [45] used three different models, i.e., K-nearest neighbor (KNN), SVM, and soft independent modeling of class analogy (SIMCA), to analyze data from 386 patients with or without CKD for clinical risk assessment and achieved accuracies over 93%. In their study, KNN and SVM achieved better performance than SIMCA. Almansour et al. [46] studied data from 400 patients with the goal of diagnosing CKD at an early stage and found that artificial neural networks (accuracy: 99.75%) performed better than SVMs (accuracy: 97.75%).

Although several studies have developed machine-learning models to detect diabetes and diabetic complications, to date, only one machine learning model has been developed to detect renal function progression in diabetic patients. Makino et al. [13] conducted a longitudinal data analysis with big data representing diabetes patients with stage 1 to 2 diabetic nephropathy and found that logistic regression models can predict DKD aggravation with 71% accuracy. A higher risk of hemodialysis was associated with DKD aggravation than with nonaggravation. However, the study was limited to the early stage of DKD and a single machine learning model with logistic regression. In our study, we found that the machine learning XGBoost model predicted the risk of developing ESRD, achieving an AUC value of 0.953 on the testing dataset.

With a positive SHAP value, the machine learning models revealed that baseline serum creatinine showed the greatest impact on predicting the risk of developing ESRD. A previous study found that better baseline renal function was protective against renal function decline [47]. Our models also found that mean serum creatinine within 1 year before diagnosis of T2DM was an important predictor of developing ESRD. The possible explanation may be that mean serum creatinine is reflective of the usual renal status. According to the SHAP dependence plots, the interaction with high-sensitivity C-reactive protein increases the prediction of risks of developing ESRD. Elevated high-sensitivity C-reactive protein was found to be independently associated with an increased risk of renal function decline in patients with diabetes and the general non-diabetic population [48, 49]. Higher UPCR levels at the time of diagnosis of T2DM were also associated with higher risks of developing ESRD, which was similar to previous research that found a positive correlation between UPCR and ESRD [50]. In contrast, female gender was associated with lower SHAP values and decreased risks of developing ESRD. A previous study also found that renal function decline in women was slower compared to men among middle-aged and elderly individuals [51].

Our study has several strengths. We established a predictive model by inputting big EMR data into the machine learning algorithm. The novelty of this study is the use of a 10-year longitudinal cohort to predict the risk of developing ESRD in newly diagnosed T2DM patients with baseline median creatinine of 0.94 mg/dL. The machine learning algorithm compared discriminative ability among different machine learning models and selected the best models. This approach offers not only improvement in AUCs but also selection of the best predicting model in cases where it is unclear what machine learning models are most suitable. In addition, the SHAP algorithm was used to interpret the model predictions, and the impacts of important features on developing ESRD were explored. Using SHAP summary plots, we demonstrated the strength and direction of each feature (positive or negative effects).

Our study also has real and perceived limitations. First, as patient information, including demographic data, underlying comorbidities and concomitant medications, was obtained from electronic health record systems and coding procedures, we could not identify mild diseases without coding in T2DM patients. Second, the inclusion of data on the duration and frequency of laboratory visits was not uniform but varied among patients. Finally, the training data and testing data were from the same dataset. Further validation in other cohorts is necessary.

## Conclusion

Our machine learning models employing longitudinal data from electronic health records were effective in predicting the risks of developing ESRD in T2DM patients in real-world clinical scenarios over a 10-year study period of observation. In addition, we used the SHAP method to provide explanations for the selected features to interpret model predictions. The developed model has the potential to predict the T2DM patients at increased risks for developing ESRD and thus, consequently initiating prevention or treatment plans for patients. In the future, external validation studies are necessary to convenient machine learning models to be developed for widespread use in clinical practice.

## Supplementary Information

**Additional file 1: Supplementary Table 1.** The performance of machine learning models after data augmentation for predicting the risk of end-stage renal disease in newly diagnosed type 2 diabetes mellitus. **Supplementary Figure 1.** Area under the receiver operating characteristic curve for the 5-fold cross-validation of the XGBoost machine learning models.

## Data Availability

The datasets generated and/or analysed during the current study are available from the corresponding author on reasonable request.
