# Supplementary material for: Prediction of the risk of developing end-stage renal diseases in newly diagnosed type 2 diabetes mellitus using artificial intelligence algorithms
Source: BioData Min. 2023 Mar 10;16:8. doi: 10.1186/s13040-023-00324-2 (PMC10007785; doi:10.1186/s13040-023-00324-2)

Prediction of the risk of developing end-stage renal diseases in newly diagnosed type 2 diabetes mellitus using artificial intelligence algorithms

Shuo-Ming Ou, MD, PhD[1,2,3], Ming-Tsun Tsai, MD, PhD[1,2,3], Kuo-Hua Lee, MD[1,2,3], Wei-Cheng Tseng, MD, PhD[1,2,3], Chih-Yu Yang, MD, PhD[1,2,3], Tz-Heng Chen, MD[1,2,3], Pin-Jie Bin[4], Tzeng-Ji Chen, MD, PhD[2,5], Yao-Ping Lin, MD, PhD[1,2,3], Wayne Huey-Herng Sheu, MD, PhD[2,6], Yuan-Chia Chu, PhD[7,8,9#], Der-Cherng Tarng, MD, PhD[1,2,3,10#]

[1]Division of Nephrology, Department of Medicine, Taipei Veterans General Hospital, Taipei, Taiwan

[2]School of Medicine, College of Medicine, National Yang Ming Chiao Tung University, Taipei, Taiwan

[3]Institute of Clinical Medicine, National Yang Ming Chiao Tung University, Taipei, Taiwan

[4]Graduate Institute of Medicine, College of Medicine, Kaohsiung Medical University, Kaohsiung, Taiwan

[5]Department of Family Medicine, Taipei Veterans General Hospital, Taipei, Taiwan

[6]Department of Internal Medicine, Taipei Veterans General Hospital, Taipei, Taiwan

[7]Information Management Office, Taipei Veterans General Hospital, Taipei, Taiwan

[8]Big Data Center, Taipei Veterans General Hospital, Taipei, Taiwan

[9]Department of Information Management, National Taipei University of Nursing and Health Sciences, Taipei, Taiwan

[10]Department and Institute of Physiology, National Yang Ming Chiao Tung University, Taipei, Taiwan

[#]These authors were co-corresponding authors.

Address correspondence and reprint requests to:

Yuan-Chia Chu, PhD

Information Management Office, Taipei Veterans General Hospital, Taipei, Taiwan

201, Section 2, Shih-Pai Road, Taipei 11217, Taiwan

Phone: 886-2-2875-7264

Fax: 886-2-2875-7136

Email: xd.yuanchia@gmail.com

and

Der-Cherng Tarng, MD, PhD

Institutes of Physiology and Clinical Medicine, National Yang Ming Chiao Tung University, Taipei; and Division of Nephrology, Department of Medicine, Taipei Veterans General Hospital, Taipei, Taiwan

201, Section 2, Shih-Pai Road, Taipei 11217, Taiwan

Phone: 886-2-2875-7517

Fax: 886-2-2875-7841

Email: dctarng@vghtpe.gov.tw

Table of Contents

**Supplemental Tables**	
Supplementary Table 1. The performance of machine learning models after data augmentation for predicting the risk of end-stage renal disease in newly diagnosed type 2 diabetes mellitus	3
**Supplemental Figures**	
Supplementary Figure 1. Area under the receiver operating characteristic curve for the 5-fold cross-validation of the XGBoost machine learning models	4

Supplementary Table 1. The performance of machine learning models after data augmentation for predicting the risk of end-stage renal disease in newly diagnosed type 2 diabetes mellitus

Model name	AUC	Accuracy	F1 score	Precision	Recall	A.precision	Sensitivity	Specificity	AUC PRC	Log loss
**Logistic regression**	0.790	0.724	0.729	0.712	0.746	0.775	0.701	0.746	0.792	0.585
**Extra trees**	0.978	0.929	0.927	0.949	0.906	0.980	0.951	0.906	0.951	0.226
**Random forest**	0.969	0.907	0.903	0.934	0.875	0.972	0.938	0.875	0.935	0.248
**GBDT**	0.978	0.929	0.928	0.944	0.912	0.979	0.946	0.912	0.950	0.197
**XGBoost**	0.988	0.953	0.953	0.962	0.944	0.990	0.963	0.944	0.967	0.134
**LGBM**	0.964	0.907	0.904	0.929	0.881	0.968	0.933	0.881	0.935	0.266

Abbreviations: AUC, area under curve of receiver operating characteristic curve; A.precision, average precision; AUC PRC, area under curve of precision-recall curve; GBDT, gradient boosting decision tree; XGBoost, extreme gradient boosting; LGBM, light gradient boosting machine.

Supplementary Figure 1. Area under the receiver operating characteristic curve for the 5-fold cross-validation of the XGBoost machine learning models

Abbreviations: XGBoost, extreme gradient boosting; ROC, receiver operating characteristic; AUC, area under the receiver operating characteristic curve; Std. dev., standard deviation;

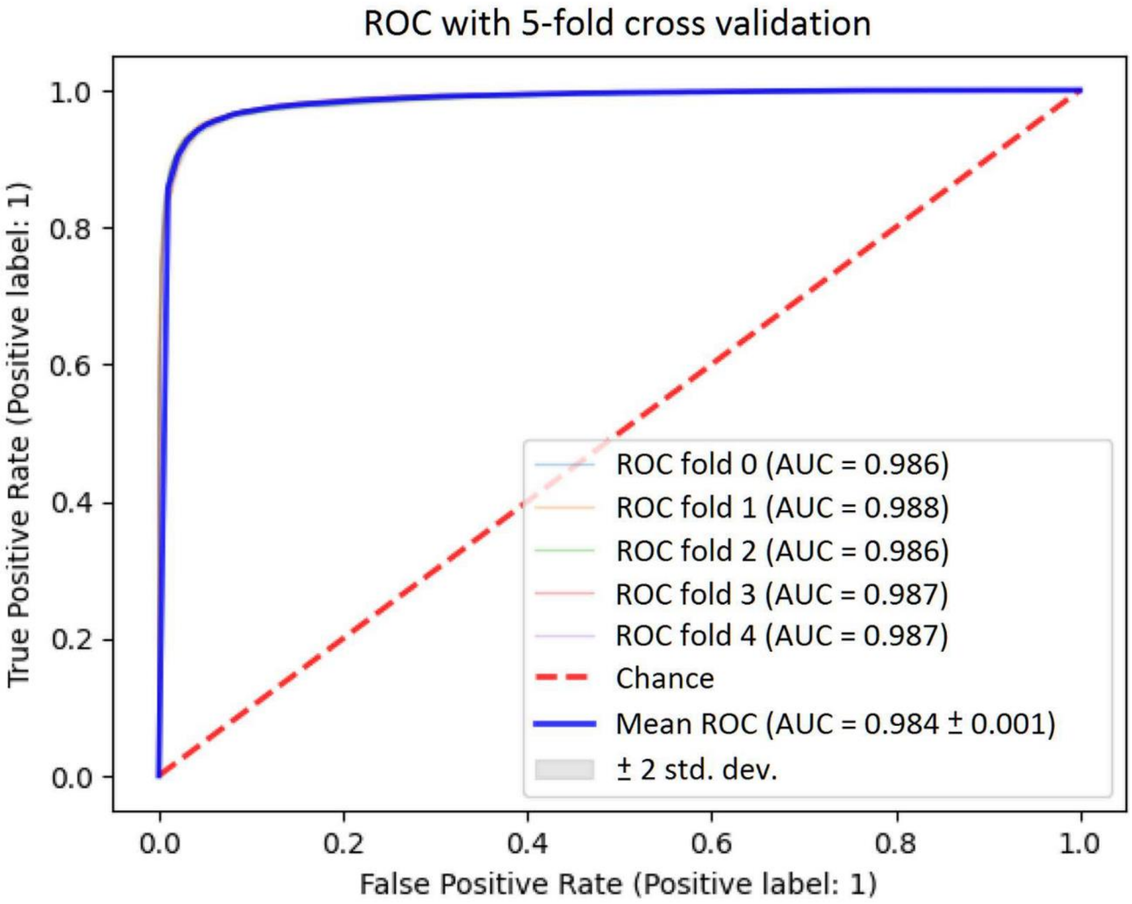

Supplement: Supplementary file 1 — Additional file 1: Supplementary Table 1. The performance of machine learning models after data augmentation for predicting the risk of end-stage renal disease in newly diagnosed type 2 diabetes mellitus. Supplementary Figure 1. Area under the receiver operating characteristic curve for the 5-fold cross-validation of the XGBoost machine learning models. [file 13040_2023_324_MOESM1_ESM.pdf]
